# Retraction Note: BMP2 secretion from hepatocellular carcinoma cell HepG2 enhances angiogenesis and tumor growth in endothelial cells via activation of the MAPK/p38 signaling pathway

**DOI:** 10.1186/s13287-022-02841-z

**Published:** 2022-04-08

**Authors:** Peng-Cheng Feng, Xing-Fei Ke, Hui-Lan Kuang, Li-Li Pan, Qiang Ye, Jian-Bing Wu

**Affiliations:** Department of Oncology, The Second Affiliated Hospital of Nanchang University, No. 1, Minde Road, Donghu District, Nanchang, 330006 Jiangxi Province People’s Republic of China

## Retraction Note: Stem Cell Research & Therapy (2019) 10:237 https://doi.org/10.1186/s13287-019-1301-2

The Editors-in-chief have retracted this article. After publication, concerns were raised regarding the validity of the presented data.

Specifically:

- Duplicated images are presented in Fig. 3g (H_shNC) and Fig. 7g (H-oeBMP2 + SB-239063);
- Duplicated images are presented in Fig. 4d (H_shNC) and Fig. 6d (H_oeBMP2 + SB-239063);
- Breaks appear to be present in the western blot backgrounds in Fig. 5a p-ERK1/2, p-JNK and JNK images, and the authors have been unable to provide the underlying raw data to address this;
- One of the tumor images in Fig. 3b (H_oeBMP2) appears highly similar to that presented in Fig. 7a (shALX4) in [1].

The Editors-in-Chief therefore no longer have confidence in the presented data and the conclusions drawn.

The authors have not responded to correspondence from the editor or publisher about this retraction.
